# *Gastrodia elata* rhizoma ameliorates thioacetamide-induced liver injury in dogs

**DOI:** 10.5455/javar.2023.j663

**Published:** 2023-06-30

**Authors:** Hye-Bin Yoon, Jeong-Hwi Cho, Jung-Soo Kim, Jun-Hee Kim, Hong-Geun Oh, Chang-Su Kim, Young-Eun Song, Gareeballah Osman Adam, Yang-Gyu Park

**Affiliations:** 1 R&D Division, HUVET Co., Ltd., Iksan-si, Korea; 2 Jeollabuk-do Agricultural Research & Extension Services, Iksan, Korea; 3 Department of Veterinary Medicine and Surgery, College of Veterinary Medicine, Sudan University of Science and Technology, Khartoum, Sudan

**Keywords:** Liver injury, *Gastrodia elata* rhizome, nitric oxide, ALT, dog

## Abstract

**Objective:**

The study aimed to investigate the hepatoprotective effects of *Gastrodia elata* rhizome (GR) on thioacetamide (TAA)-induced liver injury in dogs. We evaluated serum biochemical and hematological parameters, with emphasis on alanine transaminase (ALT), alanine phosphates (ALP), and nitric oxide (NO) levels, in dogs with TAA-induced liver injury.

**Materials and Methods:**

The animals were divided into a control group (Con), TAA group, Silymarin group (Sil, 50 mg/kg), Gastrodia rhizome low dose (GRL) (low) + TAA, GRH (high) + TAA, and GR high-dose group (GRH) control group. GRL and GRH were given daily at 50 and 100 mg/kg, respectively. TAA was given on days 1, 4, and 7 at a dose of 300 mg/kg.

**Results:**

GR significantly reduced liver injury in treated animals, as indicated by lowered levels of ALT (about 32% at day 21 in both GRL + TAA and GRH + TAA groups), ALP (about 17% and 21% at day 21 in both GRL + TAA, GRH + TAA groups, respectively), and NO (about 36% at day 21 in both GRL + TAA, GRH + TAA groups) compared to the TAA control group. Hematological parameters showed mild changes during the experiment. High-performance liquid chromatography analysis revealed gastrodin, a major component of the GR extract, constitutes 2.6% of the extract.

**Conclusion:**

The GR demonstrated significant hepatoprotective effects against TAA-induced liver injury in dogs. The study provides evidence for the potential therapeutic use of GR in the management of liver diseases.

## Introduction

Liver diseases are a major health problem that affects millions of people and animals worldwide [1]. Among the different types of liver diseases, drug-induced liver injury (DILI) is a major cause of morbidity and mortality [2]. Thioacetamide (TAA), a potent hepatotoxin, has been widely used to induce liver injury in animal models, including dogs. Although various treatments have been developed to counteract TAA-induced liver injury, they are often associated with adverse side effects [3,4].

Hepatocellular necrosis, oxidative stress, and inflammation are known effects of liver injury due to TAA. These pathological events result in elevated levels of serum biomarkers, including alanine transaminase (ALT) and alanine phosphates (ALP), which are commonly used as indicators of liver injury in dogs [4,5].

Nitric oxide (NO) is a highly reactive molecule that plays an important role in many physiological processes, including vasodilation, neurotransmission, and immune defense [6]. In the liver, NO is involved in the regulation of hepatic blood flow, the modulation of inflammation, and the regulation of oxidative stress [7]. Additionally, NO has been shown to have both pro- and anti-inflammatory effects in the liver, depending on the context and the concentration of NO [8]. Therefore, the measurement of NO levels in liver tissue could provide valuable insights into the pathogenesis of liver injury and the potential therapeutic effects of compounds. There is no specific treatment for liver diseases, as recently reviewed by Makri et al. [9]. Clinicians use numerous drugs available clinically for the treatment of liver disorders, for instance, N-acetylcysteine for DILI [10], tenofovir for viral-induced hepatic disorders [11], and mebamoxine for liver cirrhosis [12]. Nevertheless, there is a significant need for effective treatments for liver diseases, as liver diseases are a leading cause of morbidity and mortality worldwide [11,13]. Specifically, there is still a lack of effective treatments for many other liver diseases, including non-alcoholic fatty liver disease and DILI [14,15]. Multitude studies investigated the use of nutritional- and herbal-based recipes for the treatment of liver injury in animal models, for example, berberine [16], silymarin [17], *Curcuma longa* [18], etc. The majority of these studies have not yet been applied clinically.

*Gastrodia elata* rhizoma (GR), a traditional Chinese medicine, has been used for centuries to treat various ailments, including liver diseases [19]. GR contains a variety of bioactive compounds, such as gastrodin (GR) and parishin, which have been shown to possess antioxidant, anti-inflammatory, and hepatoprotective properties [20,21]. Limited research efforts examining the possible healing properties of GR in regards to liver damage, such as injury caused by medication in rodents [22,23]. However, to the best of our knowledge, no studies have evaluated the effects of GR on TAA-induced liver injury in dogs. Therefore, the objective of this study is to investigate the potential hepatoprotective effects of GR, as a plant-based alternative therapy, on TAA-induced liver injury in canines. Specifically, we aim to evaluate serum liver enzymes and NO activities in dogs with TAA-induced liver injury, with or without treatment with GR extract.

## Materials and Methods

### GR extract

The GR plant used in this study was procured from a specialized herbal market located in Muju, South Korea. The GR plant used in this study was procured from Muju Agriculture Cooperatives in Muju-gun, South Korea.

The plant material was dried, ground, and weighed. In summary, 3.3 kg of GR powder was subjected to extraction with distilled water (DW) at a ratio of 1:15. The extraction was carried out by boiling the plant material at 90°C for 6 h, followed by cooling to 65°C, and repeating the process twice. The resulting extract was concentrated using a rotary evaporator under pressure, and the remaining portion was freeze-dried to obtain a powder form. Before use, the extract was dissolved in DW.

### High-performance liquid chromatography (HPLC) analysis

In this study, we analyzed the chemical composition of a GR powder sample using an HPLC method. Specifically, 10 ml of 70% methanol was added to 1.0 gm of the GR powder sample, which was then sonicated at room temperature for 30 min, followed by centrifugation at 4,500 rpm for 20 min. The resulting supernatant was concentrated under reduced pressure and then diluted with 70% methanol to ensure that each component was within the range of the calibration curve. The diluted sample was filtered through a 0.45-um membrane filter before being analyzed by HPLC. An Agilent Zorbax Eclipse XDB C18 column (4.5 × 250 mm, 5 μm) was used in conjunction with a photodiode array detector (set to 220 nm). For the solvent, we used a 0.1% formic acid aqueous solution as solvent A and methanol containing 0.1% formic acid as solvent B. The gradient conditions were as follows: 95% (A) and 5% (B) from 0 to 5 min, 85% (A) and 15% (B) from 5 to 10 min, 45% (A) and 55% (B) from 10 to 30 min, and 95% (A) and 5% (B) from 30 to 42 min. The flow rate was set to 0.8 ml/min, and the injection amount was 10 μl. Finally, each component was quantified using an absolute calibration curve, which was generated by drawing a calibration curve with a concentration range of 12.5 to 1,000 ug/ml using a standard product (GR, 4-hydroxybenzyl alcohol). Our results show that this HPLC method is effective in determining the chemical composition of GR powder samples.

### Animals

**Figure 1.**
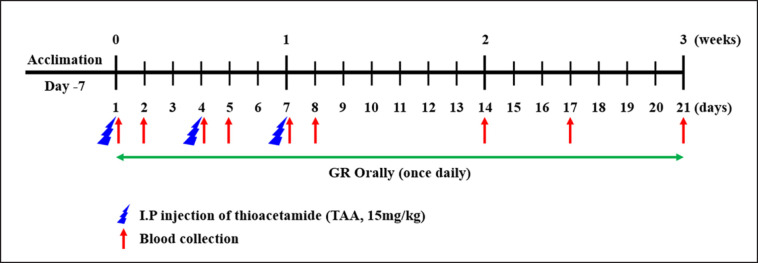
Schematic diagram of the experimental protocol.

Beagle dogs were used in this study. After acclimation, the animals were divided into six groups, as shown in Figure 1:1) the control (Con) group, received distilled water; 2) TAA groups received only TAA; 3) the Silymarin (Sil) group, given Sil and TAA; 4) GRL + TAA, received a daily dose of GR extract at a dose of 50 mg/kg and TAA; 5) GR high-dose group (GRH) + TAA, received a daily dose of 100 mg/kg GR extract plus TAA; 6) GRH control group, received only GR extract at a dose of 100 mg/kg. TAA were given intraperitoneally (IP) at a dose of 300 mg/kg at days 1, 4, and 7 based on a preliminary test done in our laboratory, which proved to be a protocol-induced liver injury. All experiments were performed with approval from the animal care committee of HUVET (HV 2022-014).

### Blood analysis

Blood samples were collected on days 1, 7, 14, and 21. Hematological parameters, including white blood cells (WBCs) and hemoglobin (HB), using the Samsung LABGEOHC10 hematology analyzer (Seoul, South Korea). ALT, ALP, total protein (T-PRO), albumin (ALB), total cholesterol (T-CHO), calcium (Ca^2 +^), creatinine (CRE), and blood urea nitrogen (BUN) were measured using an automatic biochemical analyzer (7090 Hitachi, Japan). The levels of NO were measured using an ELISA kit (BioSource, USA), according to the manufacturer’s protocol.

### Statistical analysis

The data were presented as mean ± standard deviation. The data were analyzed using one-way analysis of variance (ANOVA) using graph prism 8.0. A *p* value less than 0.05 was set as significant.

## Results

### HPLC analysis

**Figure 2.**
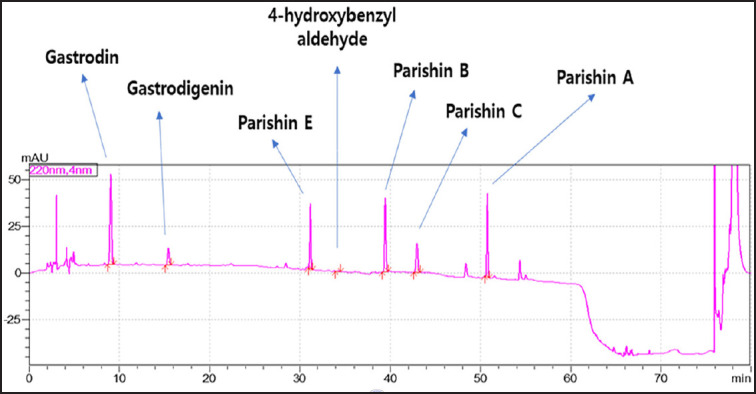
HPLC chromatogram of the *Gastrodia rhizoma* extract active components. The result indicates that the extract contains GR3.0%.

To determine the active components of the GR extract used in this study, the GR extract was analyzed using HPLC, as shown in Figure 2. Additionally, the percentage of the active components revealed the following percentages: GR 2.571%, parishin B 2.014%, parishin E 1.351, parishin A 1.035, parishin C 0.810, and gastrodignin 0.0499%.

### Effects of GR extract treatments on serum liver enzymes

**Figure 3.**
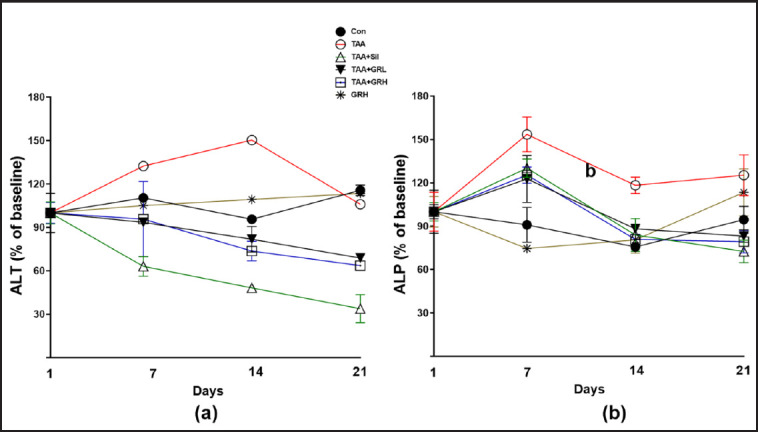
The trend of liver enzymes during the experiment. Control group (Con), thioacetamide (TAA), silymarin (Sil), *Gastrodia rhizome* low dose (GRL), and GRH represent *Gastrodia rhizome* high doses. Data are presented as a percentage of the baseline on a seven-day interval. ALT, alanine transferase; ALP, alanine phosphates.

To test the effects of GR treatments on liver function, the levels of ALT and ALP were measured in the serum (Fig. 3). The percentage of the ALT levels revealed a significant increase in the TAA group (32%, 50%, and 5% on days 7, 14, and 21, respectively) versus the Con group, while GRL and GRH indicated a substantial reduction in the percentage of the ALT levels compared with that of the TAA group as follows: 7 and 5, 19 and 27, 32, and 37%, on days 7, 14, and 21, respectively. Besides, the GR control group insignificantly elevated the levels of ALT by 5, 9, and 13% compared with that of the TAA group, whereas silymarin significantly declined the levels of ALT by 37, 52, and 67% on days 7, 14, and 21, respectively.

The changes in percentage of the ALP levels revealed a significant increase in the TAA group (53%, 18%, and 25% on days 7, 14, and 21, respectively) versus Con group, while GRL and GRH indicated an increase in the percentage of the ALP levels compared with that of the TAA group on days 7 as follows: 22 and 25, while significantly reduced on days 14 and 21 as follows: 12 and 20, 17 and 21%, respectively. Besides, the GR control group insignificantly reduced the levels of the ALP by 36, 20, but increased them by 13% on days 7, 14, and 21 compared with those of the TAA group. Although the silymarin-treated group showed an increase in the percentage of the levels of the ALP by 30% on day 7, this was later reduced by 17 and 28% on days 14 and 21, respectively.

### Effects of GR extract treatments on NO production

**Figure 4.**
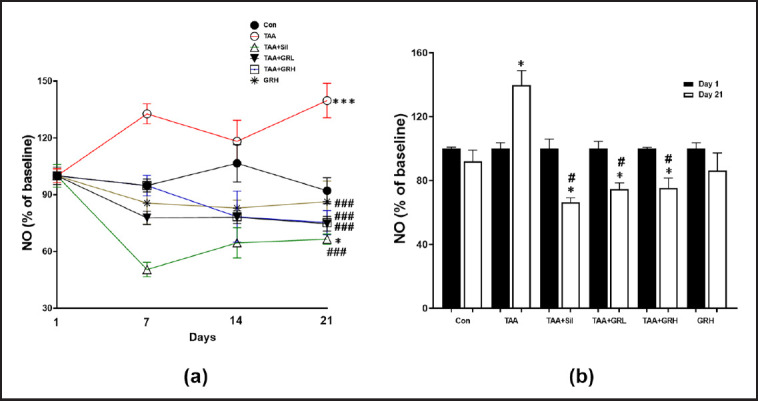
NO trend during the experiment. a) Data are presented as a percentage of the baseline on a seven days interval. The final day (Day 21) data (mean ± standard error of the mean (SEM)) were measured using one-way analysis of variance (ANOVA) followed by Tukey’s *post hoc* test. *p < 0.05 against control group (Con) and ^#^*p* < 0.05 against thioacetamide (TAA) group. b) NO% of the baseline on day 1 versus day 21. The data are reported as the mean ± SEM. *p < 0.05 (day 1 *vs*. day 21) and ^#^*p* < 0.05 versus TAA on day 21 paired Student’s *t-test*. Sil, silymarin; GRL, *Gastrodia rhizome* low dose; GRH, *Gastrodia rhizome* high dose.

NO production throughout the experiment was measured to test the effects of GR on liver injury (Fig. 4). NO% was significantly increased in the TAA group compared with the control group by 32%, 18%, and 39% on days 7, 14, and 21, respectively. On the other side, the levels of NO% were significantly reduced in the GRH + TAA and GRL + TAA groups versus TAA group as follows: 33 and 6%, 22 and 22%, 26, and 25% on days 7, 14, and 21, respectively. Sil + TAA and the GR control group showed an eventual decrease in the levels of the percentage of NO compared to that of the TAA group as follows: 50 and 15, 36 and 18, and 34 and 14% on days 7, 14, and 21, respectively. This result indicates that GR extract is safe in dogs and possesses anti-inflammatory effects against TAA-induced liver injury.

### Effects of GR extract treatments on blood and biochemical analytes

To explore the effects of the GR treatments on blood, serum parameters, and kidney function in dogs exposed to TAA, WBCs, HB, T-PRO, ALB, T-CHO, Ca^2 +^, CRE, and BUN were analyzed, as shown in Table 1. The levels of all parameters remained within the normal range except for the HB concentration in GRH, which significantly increased compared with that of the TAA group, which, although significant, is considered a minor change of 16.65 to 16.40 in average. T-CHO levels were significantly elevated in TAA, TAA + Sil, TAA + GRL, and TAA + GRH compared with the control group. However, the GRH-only treated group showed no significant change in the levels of T-CHO, which were similar to those of the control group. On the other hand, although the levels of T-CHO in all groups except GRH, which showed a significant decrease, declined, the change is still insignificant. The levels of CRE did not significantly change in all groups except in the TAA + GRH, which eventually dropped versus control and TAA groups. However, the levels of BUN significantly increased in the TAA group compared with those of the control group. However, the low dose of GR treatments eventually normalized the levels of the BUN compared with those of the TAA group. This result indicates that the GR treatments caused no toxic signs at their highest dose, and the GR treatments corrected the slight renal deterioration.

## Discussion

The current study was undertaken to investigate the effects of GR on TAA-induced liver injury in beagles’ dogs. The result indicates that GR at high doses is safe and substantially improves the liver injury induced by TAA in dogs. The levels of serum ALT, ALP, and NO in dogs with TAA-induced liver injury were substantially inhibited, which is comparable with those of the silymarin group.

Our results are consistent with previous studies that have reported the hepatoprotective effects of GR against liver injury induced by various toxins. For example, a study by Ma et al. [24] showed that GR treatment reduced serum ALT and ALP levels and attenuated liver fibrosis in rats with tetrachloride-induced liver injury [24]. Similarly, Kim and co-workers reported that GR treatment reduced serum ALT and AST levels and improved liver histology in mice with acetaminophen-induced liver injury [23]. Indeed, these chemicals damage hepatocytes, causing ALP and ALT to be released into the blood [25,26]. Our study provides further evidence of the beneficial effects of GR. We found that GR was able to protect cells from damage caused by free radicals and inflammation. GR also inhibits apoptosis. These effects can be attributed to the presence of GR in GR extract [27], which is abundantly found in our extract.

In fact, the GR content of the samples analyzed in this study was higher than the GR content of samples analyzed in a recent report, which found that GR content is 3.68 ± 0.02 μg/mg [28] and better than a recent report analyzing samples from Korea, China, and Japan, which concluded that 0.37% to 0.79% is the GR content. It is important to note that the extraction method used in this study was different from those used in previous studies. In this study, we used water for extraction, while previous studies have used other organic solvents. It is possible that the use of water may have affected the concentration of GR in the extracts. It is expected that the highest content of GR results in better protective effects because pure GR has been reported to ameliorate inflammation in nerves by inhibiting inflammatory cytokines [29] and Ca^2 + ^/CaMKII signals [30] and spinal cord injury via antioxidant and anti-inflammatory effects [31].

**Table 1.** Effects of GR treatments on blood and biochemical analytes.

	Con	TAA				
			Sil	GRL	GRH	GRH
WBCs (×10^9^/l)	10.05 ± 0.17	10.05 ± 1.30	10.35 ± 4.10	9.90 ± 0.35	9.70 ± 0.12	8.35 ± 0.60
HB (%)	15.15 ± 0.29	15.65 ± 0.40	16.15 ± 0.98	16.30 ± 0.12	16.70 ± 0.58	16.40 ± 0.58^#^
T-PRO (mg/dl)	6.08 ± 0.22	6.00 ± 0.23	6.00 ± 0.12	6.15 ± 0.58	6.35 ± 0.75	5.55 ± 0.29
ALB (gm/dl)	3.17 ± 0.15	3.15 ± 0.06	3.10 ± 0.23	3.10 ± 0.08	3.05 ± 0.06	2.90 ± 0.12
T-CHO (mg/dl)	165.8 ± 5.0	209.5 ± 3.8^*^	210.5 ± 3.2^*^	205.5 ± 11.0^*^	200.5 ± 2.6^*^	179.3 ± 5.5^#^
Ca^2 + ^(mg/dl)	10.5 ± 0.6	10.9 ± 0.2	11.1 ± 0.1	10.6 ± 0.2	10.7 ± 0.2	9.9 ± 0.1^#^
CRE (mg/dl)	0.48 ± 0.03	0.51 ± 0.05	0.45 ± 0.06	0.04 ± 0.03	0.37 ± 0.05^*##^	0.51 ± 0.05
BUN (mg/dl)	10.1 ± 1.0	14.2 ± 2.9^*^	11.7 ± 1.4	10.5 ± 0.3^#^	12.6 ± 0.9	12.6 ± 0.5

Data are reported as the mean ± SDM (*n* = 5). *p < 0.05, Tukey’s *post hoc* test following one-way ANOVA versus the normal control group (Cont); ^#^*p* < 0.05, Tukey’s post *hoc test* following one-way ANOVA versus the thioacetamide (TAA) group. Silymarin (Sil, 50 mg/kg), GRL (*Gastrodia elata* rhizome low dose, 50 mg/kg), GRH (*Gastrodia elata* rhizome low dose, 100 mg/kg), blood cells (WBCs), and hemoglobin (HB), total protein (T-PRO), albumin (ALB), total cholesterol (T-CHO), calcium (Ca^2 + ^), creatinine (CRE), and blood urea nitrogen (BUN).

Serum transaminases are enzymes that are involved in the metabolism of amino acids. NO is a gas that is involved in a variety of biological processes, including vasodilation and inflammation. The lack of significant changes in serum transaminase or NO levels suggests that GR did not cause any liver damage or inflammation in dogs at high doses [32,33]. Although NO is well known for its role in the immune system and blood pressure [34], several reports indicate its role in oxidative stress in liver [35]; NO is a key mediator of liver pathophysiology induced by pro-inflammatory cytokines during the inflammatory process [36]. These findings are consistent with our result in Figure 4, where NO eventually elevated in TAA groups while GR treatments reduced NO production. In this study, the hepatoprotective effects of GR occurred via its modulatory effect on hepatic enzymes (ALP and ALT) and NO production, reducing liver injury.

Besides its toxic effects on the liver, TAA is known to cause blood abnormalities, lipoprotein abnormalities [37], and kidney disorders [38]. Contrary to the present study, studies have reported that TAA causes a decline in the levels of RBCs and HB and an increase in WBC levels due to acute inflammation in response to chemicals such as TAA [39,40].

The disturbance occurred due to the period between the last dose of TAA on day 7 and sacrifice day, to which the negligible changes in the levels of Ca and CRE can be attributed. GR antioxidant capacity can mitigate the renal damage indicated by reducing the levels of BUN [30,41]. The GR effects on lipids were unsatisfactory, although the GRH-only treated group reduced the T-CHO in rats. Nonetheless, in this study, we focused on liver injury and the protective role of the GR extracts. Hence, there is a need to address this in a separate study to understand the effects of GR on lipid metabolism in normal and liver injury models. Although the study proved that GR has significant beneficial effects on TAA-induced liver injury in dogs, there are several limitations. First, the mechanisms behind the GR effects are not fully examined, such as measuring antioxidants or signaling. Second, histology or computer tomography scans of the liver were not screened. The identified limitations will serve as the basis for the upcoming investigation. Nevertheless, the study provides evidence of protective effects against liver injury.

## Conclusion

The present study provides further evidence of the hepatoprotective effects of GR against TAA-induced liver injury in dogs and suggests that the observed effects may be mediated, at least in part, through the modulation of transaminases and NO. That being said, insights into the underlying mechanisms of GR’s hepatoprotective effects are required by exploring the genes or proteins regulated by GR or GR-purifiedGR. These findings could have important implications for the development of new treatments for liver diseases in both animals and humans.

## List of Abbreviations

ALT, Alanine transaminase; ALP, Alanine phosphates; ALB, Albumin; ANOVA, Analysis of variance; APAP, Acetaminophen; BUN, Blood urea nitrogen; Con, Control group; Ca^2+^, Calcium; CRE, Creatinine; DILI, Drug-induced liver injury; DW, Distilled water; GR, *Gastrodia elata rhizome; *GRH, GR high-dose group; GRL, GR low-dose group; HPLC, High-performance liquid chromatography; HB, Haemoglobin; NO, Nitric oxide; RBC, Red Blood Cell Sil, Silymarine group; TAA, Thioacetamide; T-PRO, Total protein; T-CHO, Total cholesterol; WBC, White blood cells
